# Identifying prognostic factors for pulmonary embolism patients with hemodynamic decompensation admitted to the intensive care unit

**DOI:** 10.1097/MD.0000000000036392

**Published:** 2024-01-19

**Authors:** Yanbin Peng, Zhong Chen, Zhongkai Luo, Gaosheng Luo, Yunfeng Chu, Bo Zhou, Siqi Zhu

**Affiliations:** a Department of Hand Microsurgical Technique Surgery, Peking University Shenzhen Hospital, Shenzhen City, Guangdong Province, China; b Baise Tiandong County People’s Hospital, Tiandong County, Baise City, Guangxi Zhuang Autonomous Region Province, China; c Department of Orthopaedics Surgery, Baise Tiandong County People’s Hospital, Tiandong County, Baise City, Guangxi Zhuang Autonomous Region Province, China.

**Keywords:** intensive care unit, pulmonary embolism, mortality, prognostic factors

## Abstract

We aimed to determine prognostic indicators of PE patients with hemodynamic decompensation admitted to the ICU. PE patients with hemodynamic decompensation at ICU admission from Medical Information Mart for Intensive Care IV database were included. Least absolute shrinkage and selection operator with 2 specific lambdas were performed to reduce the dimension of variables after univariate analysis. Then we conducted multivariate logistic regression analysis and 2 models were built. A total of 548 patients were included, among whom 187 died. Lactate, creatine-kinase MB, troponin-T were significantly higher in death group. Eight common factors were screened out from first model statistically mostly in consistent with second model: older age, decreased hemoglobin, elevated anion gap, elevated International Standard Ratio (INR), elevated respiratory rate, decreased temperature, decreased blood oxygen saturation (SpO2) and the onset of cardiac arrest were significantly risk factors for in-Hospital mortality. The nonlinear relationships between these indicators and mortality were showed by the restricted cubic spline and cutoff values were determined. Our study demonstrated that age, hemoglobin levels, anion gap levels, INR, respiratory rate, temperature, SpO2 levels, the onset of cardiac arrest could be applied to predict mortality of PE patients with hemodynamic decompensation at ICU admission.

## 1. Introduction

Pulmonary embolism (PE) belongs to a serious kind of venous thromboembolism (VTE), is a potentially life-threating, lifelong disease. Several major adverse events could happen in patients with PE especially in the first year after diagnosis, such as death, post-PE syndrome, recurrent VTE, major bleeding related with use of anticoagulants.^[1]^ The remarkable fact is demonstrated from an Australian study that PE leads to about 11 cases of intensive care unit (ICU) admission per million people each year, nearly 5% of who die in the hospital.^[2,3]^ Given that the highest risk of PE-related death happens within the first 3 months after diagnosis, timely identification of PE-related prognosis factors could promote appropriate monitoring and treatment, which do help to reduce the incidence of adverse events.^[4]^

The 2019 European Society of Cardiology (ESC) guidelines define PE patients with hemodynamic instability (one of cardiac arrest, obstructive shock or persistent hypotension) as the high-risk group who are confronted with early death, and recommend hemodynamic status and kinds of biomarkers composing of troponin-T, N-terminal (NT)-proBNP, lactate, serum creatinine levels (and so on) are important factors associated with general PE patients’ prognosis.^[5]^ Nevertheless, variation in prognostic indicators may exist across various studies and patient subgroups. Whether the above indicators are applicable to specific PE groups such as patients with hemodynamic decompensation remains to be further verified. Hemodynamic compromise could have a great impact on the levels of numerous indicators in these specific PE patients compared with normotensive groups.^[6,7]^ For example, the severe injury of cardiomyocytes caused by imbalance between oxygen supply and demand in PE patients with hemodynamic instability could be bound up with the significant increase in circulating levels of biomarkers of myocardial injury. Furthermore, seldom study investigated the risk factors for mortality in PE patients with hemodynamic decompensation admitted to the ICU. Therefore, we conducted this retrospective study to determine the prognostic indicators which may indicate the poor prognosis in PE patients with hemodynamic decompensation admitted to the ICU.

## 2. Material and methods

### 2.1. Patients and clinical treatment

This was a retrospective study from the Medical Information Mart for Intensive Care IV (MIMIC-IV version 1.0) database from 2008 to 2019. MIMIC-IV used both International Classification of Diseases (ICD) 9 and ICD-10 code to defined diseases status and complications of individual. Through using ICD-9 of 41512 and 41519, ICD-10 of I26, I260, I2601, I2609, I269, I2690, I2693 and I2699, critical PE patients defined as pulmonary embolism with or without cor pulmonale, septic pulmonary embolism with or without cor pulmonale admitted to ICU were both included into this analysis. The diagnosis and clinical treatment flow could be referred to 2019 ESC Guidelines for the diagnosis and management of acute PE. Multidetector computer tomographic pulmonary angiography (CTPA) with clinical presentation including dyspnea, chest pain, presyncope or hemoptysis was the golden diagnoses criterion. Definition of hemodynamic decompensation including cardiac arrest or need for cardiopulmonary resuscitation, obstructive shock (including systolic blood pressure [SBP] < 90 mm Hg or vasopressors required, and end-organ hypoperfusion) and persistent hypotension (including SBP < 90 mm Hg or SBP drop > 40 mm Hg, lasting longer than 15 minutes and not caused by new-onset arrhythmia, hypovolaemia, or sepsis). For risk stratification, simplified pulmonary embolism index (sPESI), simplified acute physiology score II (SAPSII) and sequential organ failure assessment (SOFA) were calculated for every individual. Administration of supplemental oxygen was indicated in patients with SpO2 < 90%. High-flow oxygen and mechanical ventilation (noninvasive or invasive) were used to treat hypoxemia. Vasopressor use was also important treatment in hemodynamic decompensation especially congestive heart failure or cardiogenic shock and it included use of dobutamine, dopamine, epinephrine, norepinephrine, phenylephrine or milrinone. For cardiac arrest, advanced life support and thrombolytic therapy should be disposed early. Excluding criterion including age lower than 18 and not urgent or emergency admission. This study was conducted in accordance with the Declaration of Helsinki (as revised in 2013) and permission of the institutional review boards of both Beth Israel Deaconess Medical Center and Massachusetts Institute of Technology Affiliates. Requirement for patient consent was waived because this was a retrospective study and did not impact clinical practice.

### 2.2. Variables and outcome definition

From MIMIC-IV, general information, laboratory test, vital signs, complication, treatment information and severity scores were obtained. Acute kidney injury was defined based on kdigo criteria. Laboratory results were all tested within 24 hours after ICU admission, including cardiac marker, hemogram index, biochemical markers and clotting markers. Vital signs included blood pressure, heart rate, respiratory rate, temperature, SpO2 and temperature tested within 24 hours after ICU admission. In treatment information, oxygen therapy included high flow nasal cannula and mechanical ventilation (noninvasive or invasive). Urine output was the total volume within the 24 hours after ICU admission. Invasive line included both arterial and venous catheter.

Major outcome was defined as in-Hospital death. Secondary outcome was defined as in-ICU death, length of hospital stay and length of ICU stay.

### 2.3. Statistical analysis

Firstly, baseline data of patients was grouped by prognosis (in-Hospital death) and compared. Continuous variables were presented as median and interquartile range, compared with Kruskal Wallis test; Categorical variables were presented as percentage, compared with Chi-square test. Two tailed *P* value smaller than .05 was defined as statistical significance. In all continuous baseline data, missing value of body mass index (BMI), neutrophil to lymphocyte ratio, lactate, creatine-kinase creatine phosphokinase, creatine-kinase Myocardial Band (CK-MB), N-terminal prohormone of brain natriuretic peptide was higher than 15%. Among then, variables that showed significance in univariate analysis were then used to predict in-Hospital mortality and in-ICU mortality based on receiver operator curve (ROC). For other variables that had missing value lower than 15%, single imputation based on the complete conditional specification and predictive mean matching method was used to full-fill them. The imputed database was then put into logistic regression with L1 regularization (i.e. least absolute shrinkage and selection operator^[8]^). LASSO helps reduce the model dimension and the collinearity of variables by increasing the penalty coefficient lambda, and can be used to select important clinical risk factor. And we could get 2 models based on 2 special lambdas. First one made the model had minimum mean cross-validated error. Second one was the largest value of lambda that made the error was within 1 standard error of the minimum. After selecting the important variables in 2 models, they were fitted with multivariate logistic regression model to adjusted the cofounding factors. And the significance variables in the multivariate analysis were then used to predict in-Hospital and in-ICU mortality based on ROC. The prediction results of 2 models were also used to predict the outcome.

Moreover, significant continuous variables in multivariate analysis were then used to show the effects on in-Hospital mortality presented in restricted cubic splines (RCS). RCS can help to show the smooth risk (odds ratio) change of continuous variables and determine the risk cutoff value. At last, baseline data of patients was grouped by cardiac arrest and compared because cardiac arrest was the major lethal factor for critical PE.

## 3. Results

### 3.1. Enrollment

Based on the enrollment and excluding criterion, at first, 1573 PE patients were enrolled from MIMIC-IV and then 46 patients were excluded because they were not urgent or emergency admission. Among the rest, 547 patients (35.8%) were defined as hemodynamic decompensation. And the baseline data of their first ICU admission were used for further analysis.

### 3.2. Baseline data compare

When grouped with in-Hospital death, death group showed higher age (*P* < .001), more severe kidney injury stage (*P* < .001), higher lactate (*P* < .001), higher CK-MB (*P* = .011), higher Troponin-T (*P* = .005), higher WBC (*P* = .026), higher anion gap (*P* < .001), lower bicarbonate (*P* < .001), higher BUN (*P* < .001), higher creatinine (*P* < .001), higher INR (*P* < .001), higher PT (*P* < .001), higher PTT (*P* < .001), lower temperature (*P* < .001), lower SpO2 (*P* < .001), higher glucose (*P* < .001), more septicemia (*P* = .03), more atrial fibrillation (*P* = .001), more metastatic solid tumor (*P* = .046), more sinus tachycardia (*P* = .011), lower urine output in first day (*P* < .001), more invasive ventilation (*P* = .007), higher SAPSII (*P* < .001), SOFA (*P* < .001) and sPESI (*P* < .001), more cardiac arrest (*P* < .001) and more vasopressor use (*P* < .001). More result was shown in Table 1.

**Table 1:** Baseline data and outcome of hemodynamic decompensation pulmonary embolism patients stratified by prognosis

Variables	Survival (N = 360)	Death (N = 187)	*P* value	Missing value
General information				
Male	184 (51.1%)	85 (45.5%)	.244	0
Age (years)	65.01 [53.18, 75.16]	71.16 [59.42, 80.15]	**<.001**	0
BMI (kg/m^2^)	28.00 [24.00, 33.60]	28.90 [25.00, 34.00]	.486	172
Proximal DVT	33 (9.2%)	13 (7.0%)	.47	0
VTE history	7 (1.9%)	7 (3.7%)	.328	0
Length of hospital stay (days)	17.35 [10.82, 27.76]	8.07 [2.18, 15.65]	**<.001**	0
Length of ICU stay (days)	5.80 [2.92, 12.88]	4.74 [1.44, 10.96]	**.002**	0
Acute kidney injury	303 (84.2%)	166 (88.8%)	.183	0
Kidney injury stage			**<.001**	0
0	57 (15.8%)	21 (11.2%)		
1	50 (13.9%)	16 (8.6%)		
2	145 (40.3%)	38 (20.3%)		
3	108 (30.0%)	112 (59.9%)		
Laboratory test				
NLR	9.79 [5.93, 17.48]	12.00 [5.94, 21.66]	.259	150
Lactate (mmol/L)	2.40 [1.48, 3.92]	3.80 [1.98, 8.17]	**<.001**	107
CK-CPK (U/L)	189.00 [63.00, 577.25]	234.50 [74.75, 699.00]	.498	305
CK-MB (U/L)	5.00 [3.00, 10.00]	7.00 [3.00, 24.00]	**.011**	284
Troponin-T (μg/L)	0.07 [0.03, 0.20]	0.15 [0.04, 0.32]	**.005**	301
NT-proBNP (pg/mL)	2763.00 [1190.25, 7749.75]	4685.00 [1452.00, 14756.00]	.097	404
Hematocrit (%)	35.00 [30.30, 40.30]	34.00 [28.90, 39.90]	.351	0
Hemoglobin (g/dL)	11.30 [9.70, 13.20]	11.05 [9.10, 12.78]	.099	1
Platelets (K/μL)	219.00 [153.00, 295.50]	209.00 [135.00, 292.50]	.236	1
WBC (K/μL)	14.95 [10.60, 20.15]	16.65 [11.17, 23.17]	**.026**	1
Anion Gap (mEq/L)	16.00 [13.00, 20.00]	19.00 [15.25, 23.00]	**<.001**	1
Bicarbonate (mEq/L)	24.00 [21.00, 26.00]	22.00 [19.00, 25.00]	**<.001**	1
BUN (mg/dL)	23.00 [15.00, 34.50]	31.00 [21.00, 48.00]	**<.001**	1
Creatinine (mg/dL)	1.00 [0.80, 1.60]	1.40 [1.00, 2.40]	**<.001**	0
INR	1.40 [1.20, 1.70]	1.50 [1.30, 2.15]	**<.001**	19
PT (s)	15.10 [13.30, 18.40]	16.85 [14.10, 23.33]	**<.001**	18
PTT (s)	38.00 [29.30, 87.60]	64.95 [32.18, 150.00]	**<.001**	18
Vital signs				
Heart rate (1/min)	115.00 [97.00, 130.00]	114.00 [102.00, 132.75]	.511	1
SBP (mmHg)	142.00 [127.50, 158.00]	143.00 [127.00, 157.00]	.696	3
DBP (mmHg)	85.00 [74.50, 99.00]	87.00 [77.00, 102.00]	.194	3
MBP (mmHg)	101.00 [89.00, 117.00]	101.00 [91.00, 113.75]	.856	1
Respiratory rate (1/min)	13.00 [11.00, 15.25]	14.00 [10.25, 17.00]	.121	1
Temperature (°C)	37.47 [37.06, 38.22]	37.22 [36.94, 37.94]	**<.001**	28
SpO_2_ (%)	92.00 [90.00, 95.00]	90.00 [83.00, 93.00]	**<.001**	1
Glucose (mg/dL)	160.00 [127.00, 212.00]	185.00 [145.50, 311.25]	**<.001**	7
Complication				
Septicemia	61 (16.9%)	47 (25.1%)	**.03**	0
Congestive heart failure	119 (33.1%)	67 (35.8%)	.579	0
Hypertension	151 (41.9%)	86 (46.0%)	.415	0
Stroke	26 (7.2%)	17 (9.1%)	.547	0
Atrial fibrillation	99 (27.5%)	78 (41.7%)	**.001**	0
Chronic pulmonary disease	104 (28.9%)	54 (28.9%)	1	0
Myocardial infarct	62 (17.2%)	41 (21.9%)	.223	0
Malignant cancer	64 (17.8%)	46 (24.6%)	.076	0
Metastatic solid tumor	40 (11.1%)	33 (17.6%)	**.046**	0
Severe liver disease	6 (1.7%)	7 (3.7%)	.224	0
Sinus tachycardia	285 (79.2%)	129 (69.0%)	**.011**	
Treatment				
Urine output in first day (mL/d)	1453.00 [920.00, 2160.00]	819.50 [388.50, 1473.75]	**<.001**	24
Oxygen therapy			**.007**	0
None	16 (4.4%)	12 (6.4%)		
Non-invasive	103 (28.6%)	31 (16.6%)		
Invasive	241 (66.9%)	144 (77.0%)		
Invasive line	318 (88.3%)	173 (92.5%)	.167	0
Severity score				
SAPSII	41.27 (13.71%)	53.06 (16.32%)	**<.001**	0
SOFA	7.56 (4.16%)	9.76 (4.15%)	**<.001**	0
sPESI	2.46 (1.07%)	2.93 (1.04%)	**<.001**	3
Reasons for hemodynamic decompensation				
Cardiac arrest	3 (0.8%)	15 (8.0%)	**<.001**	0
Obstructive shock	51 (14.2%)	33 (17.6%)	.344	0
Persistence hypotension	151 (41.9%)	78 (41.7%)	1	0
Vasopressor	298 (82.8%)	176 (94.1%)	**<.001**	0

Bold values mean statistical significance for *P* values.

CK-CPK = creatine-kinase creatine phosphokinase, CK-MB = creatine-kinase MB, MBP = mean blood pressure, NLR = neutrophil to lymphocyte ratio, NT-proBNP = N-terminal prohormone of brain natriuretic peptide, PTT = partial thromboplastin time, SpO_2_ = percutaneous oxygen saturation, SAPSII = simplified acute physiology score II, SBP = systolic blood pressure, SOFA = sequential organ failure assessment, sPESI = simplified pulmonary embolism severity index, VTE = venous thromboembolism.

Continuous variables were presented as median and interquartile range, compared with Kruskal Wallis test; Categorical variables were presented as percentage, compared with Chi-square test. Two tailed *P* value smaller than .05 was defined as statistical significance.

### 3.3. Variables selection and model fit

**Figure 1.**
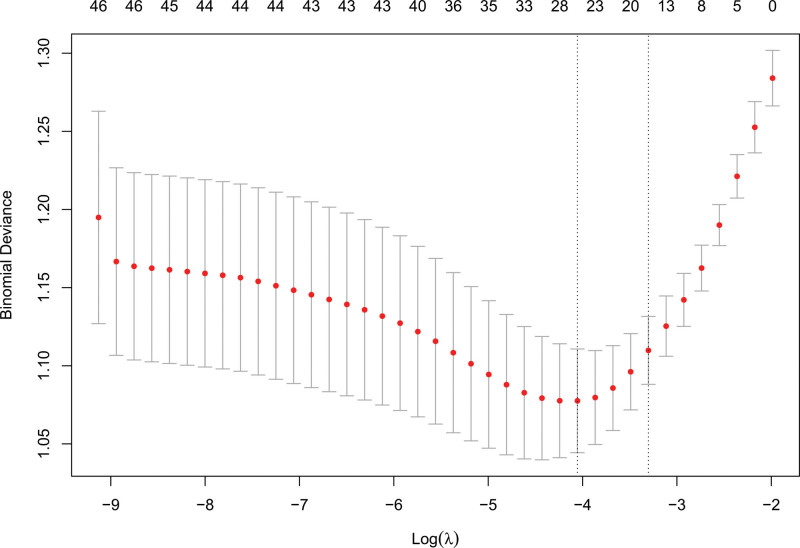
Use cross validation of LASSO analysis to select candidate variables. As lambda in LASSO increased, the variables in regression decreased and the binomial deviance firstly decreased and then increased. Left dotted line means the model based on value of lambda that gave minimum mean cross-validated error (First model). Right dotted line means the model based on largest value of lambda such that error was within 1 standard error of the minimum (Second model). Line segment of each model represents 95% CI of binomial deviation. CI = confidence interval, LASSO = Least absolute shrinkage and selection operator.

Based on cross validation of LASSO analysis (Fig. 1), 2 models were built. 23 variables including age, VTE history, kidney injury stage, hemoglobin, platelets, WBC, anion gap, bicarbonate, INR, PTT, respiratory rate, temperature, SpO2, septicemia, stroke, atrial fibrillation, malignant cancer, metastatic solid tumor, severe liver disease, urine output, oxygen therapy, cardiac arrest and vasopressor, were included in the first model. Among then, 10 variables including age, hemoglobin, anion gap, INR, respiratory rate, temperature, SpO2, stroke, metastatic solid tumor and cardiac arrest showed statistical significance (*P* < .05). In the second model, 15 variables including age, kidney injury stage, hemoglobin, anion gap, INR, PTT, temperature, SpO2, atrial fibrillation, malignant cancer, metastatic solid tumor, urine output, oxygen therapy, cardiac arrest and vasopressor were included. And only 6 variables including hemoglobin, anion gap, INR, temperature, SpO2 and cardiac arrest showed statistical significance (*P* < .05) (Table 2).

**Table 2:** Variables and coefficients in two multivariable models selected with LASSO

Variables	First model			Second model		
	OR (95%CI)	Standard error	*P* value	OR (95%CI)	Standard error	*P* value
Age	1.016 (1.001–1.033)	0.008	**.042**	1.011 (0.996–1.026)	0.008	.164
VTE history	2.360 (0.614–9.016)	0.677	.205	NA	NA	NA
Kidney injury stage						
0	1 (Reference)	NA	NA	1 (Reference)	NA	NA
1	0.914 (0.346–2.395)	0.491	.855	0.873 (0.345–2.19)	0.469	.772
2	0.818 (0.367–1.873)	0.414	.626	0.792 (0.373–1.732)	0.390	.550
3	2.114 (0.966–4.793)	0.407	.066	2.026 (0.964–4.404)	0.386	.067
Hemoglobin	0.890 (0.803–0.984)	0.052	**.025**	0.89 (0.805–0.982)	0.050	**.021**
Platelets	0.999 (0.997–1)	0.001	.177	NA	NA	NA
WBC	1.009 (0.997–1.03)	0.009	.354	NA	NA	NA
Anion gap	1.048 (1.005–1.094)	0.022	**.029**	1.047 (1.008–1.088)	0.019	**.017**
Bicarbonate	0.992 (0.943–1.043)	0.026	.756	NA	NA	NA
INR	1.247 (1.029–1.548)	0.101	**.028**	1.275 (1.058–1.594)	0.101	**.017**
PTT	1.004 (0.999–1.008)	0.002	.149	1.004 (0.999–1.008)	0.002	.130
Respiratory rate	1.053 (1.002–1.108)	0.026	**.043**	NA	NA	NA
Temperature	0.636 (0.487–0.818)	0.132	**.001**	0.695 (0.541–0.882)	0.124	**.003**
SpO_2_	0.978 (0.958–0.997)	0.010	**.025**	0.978 (0.959–0.996)	0.010	**.023**
Septicemia	1.493 (0.867–2.562)	0.276	.146	NA	NA	NA
Stroke	3.106 (1.411–6.782)	0.398	**.004**	NA	NA	NA
Atrial fibrillation	1.465 (0.9–2.381)	0.248	.123	1.483 (0.923–2.384)	0.242	.103
Malignant cancer	1.695 (0.915–3.123)	0.312	.091	1.551 (0.859–2.784)	0.299	.143
Metastatic solid tumor	2.021 (1.004–4.082)	0.357	**.048**	1.767 (0.898–3.489)	0.345	.099
Severe liver disease	2.70 (0.698–10.72)	0.689	.149	NA	NA	NA
Urine output on first day	0.9998 (0.9996-1.00003)	0.0001	.099	0.9998 (0.9996-1.00003)	0.0001	.094
Oxygen therapy						
None	1 (Reference)	NA	NA	1 (Reference)	NA	NA
Non-invasive	0.582 (0.182–1.934)	0.599	.366	0.615 (0.206–1.904)	0.564	.388
Invasive	1.561 (0.489–5.267)	0.603	.460	1.537 (0.515–4.793)	0.566	.447
Cardiac arrest	5.777 (1.358–33.454)	0.799	**.028**	6.77 (1.631–37.86)	0.785	**.015**
Vasopressor	1.846 (0.85–4.292)	0.410	.135	2.034 (0.966–4.617)	0.396	.073

Multivariable logistic regression was conducted to fit the models. Variables in the first model were selected with LASSO based on the value of lambda that gave minimum mean cross-validated error. Variables in the second model were selected with LASSO based on the largest value of lambda such that the error was within 1 standard error of the minimum. Two-tailed *P* value smaller than 0.05 was defined as statistical significance. Bold values mean statistical significance for *P* values.

CI = confidence interval, INR = international standard ratio, LASSO = least absolute shrinkage and selection operator, NA = not available, OR = Odds ratio, PTT = partial thromboplastin time, SpO_2_ = percutaneous oxygen saturation, VTE = venous thromboembolism, WBC = white blood cell.

### 3.4. Prediction ability

Then, significant variables in univariate analysis (lactate, CK-MB, troponin-T, missing value higher than 15%) and multivariable (imputed database) were used to predict the outcome based on ROC (Table 3). Lactate, anion gap got area under the curve (AUC) higher than 0.65 when predicting in-Hospital death. Lactate, anion gap and SpO2 got AUC higher than 0.65 when predicting in-ICU death. Fist model got an AUC of 0.840 (95% CI: 0.805–0.874) when predicting in-Hospital death, 0.842 (95% CI: 0.807–0.878) when predicting in-ICU death. Second model got an AUC of 0.821 (95% CI: 0.785–0.858) when predicting in-Hospital death, 0.835 (95% CI: 0.798–0.871) when predicting in-ICU death.

**Table 3:** AUC of prognosis factor selected in previous univariate and multivariate analysis.

Variables	Predict in-Hospital Death AUC (95% CI)	Predict in-ICU Death AUC (95% CI)
Lactate	0.673 (0.620–0.725)	0.687 (0.630–0.744)
CK-MB	0.591 (0.520–0.661)	0.591 (0.516–0.667)
Troponin-T	0.605 (0.533–0.677)	0.594 (0.517–0.670)
Age	0.605 (0.556–0.655)	0.603 (0.550–0.657)
Hemoglobin	0.546 (0.494–0.597)	0.509 (0.454–0.565)
Anion gap	0.664 (0.617–0.712)	0.698 (0.649–0.748)
INR	0.627 (0.577–0.678)	0.629 (0.571–0.686)
Respiratory rate	0.542 (0.490–0.593)	0.532 (0.475–0.589)
Temperature	0.612 (0.561–0.662)	0.631 (0.575–0.687)
SpO2	0.630 (0.580–0.681)	0.673 (0.618–0.727)
First model	0.840 (0.805–0.874)	0.842 (0.807–0.878)
Second model	0.821 (0.785–0.858)	0.835 (0.798–0.871)

AUC = area under the curve, CI = confidence interval, ICU = intensive care unit, CK-MB = creatine-kinase MB, INR = International Standard Ratio, SpO2 = percutaneous oxygen saturation.

### 3.5. Restricted cubic splines analysis

**Figure 2.**
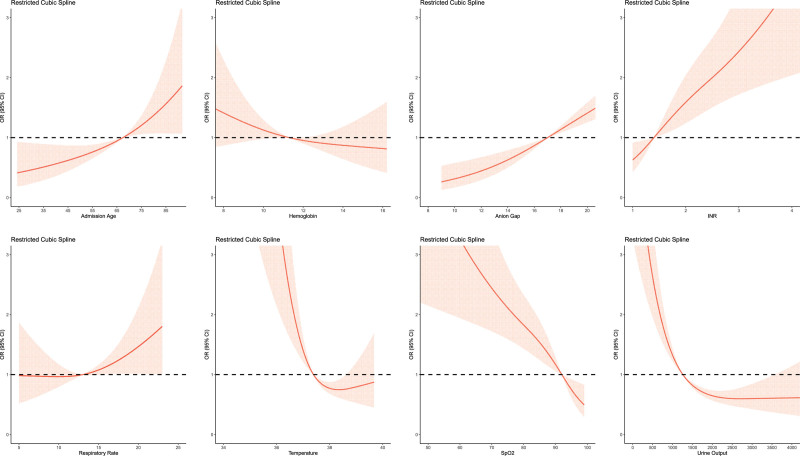
Effects of risk factors on in-Hospital mortality presented in restricted cubic splines. Restricted cubic spline graph showing the association between risk factor and in-Hospital mortality. Pink-shaded area indicates 95% CI. cutoff value for age was 67, for hemoglobin was 11.3, for anion gap was 17, for INR was 1.4, for respiratory rate was 13, for temperature was 37.3, for SpO2 was 92, for urine output was 1250. CI = confidence interval, OR = odds ratio.

Figure 2 shows the effects of risk factors on in-Hospital mortality presented in restricted cubic splines. The cutoff value could be identified when the red line contacted the horizontal dotted line that represented the odds ratio was 1. cutoff value for age was 67, for hemoglobin was 11.3, for anion gap was 17, for INR was 1.4, for respiratory rate was 13, for temperature was 37.3, for SpO2 was 92, for urine output was 1250. 4 variables including age, anion gap, INR and respiratory rate showed nearly positive association with in-Hospital mortality, which meant higher value had higher risk of mortality. Hemoglobin and SpO2 showed nearly negative association with in-Hospital morality, which meant higher value had lower risk of mortality. Temperature and urine output might show a U shape between value and the mortality. The results were all consistence with clinical experience.

### 3.6. Risk stratification by cardiac arrest

Cardiac arrest was the only categorical variable that both showed significance in 2 models and is usually the lethal factor in clinic. At last, baseline data of patients was grouped by cardiac arrest and compared, only significant variables were shown in Table 4. 15 patients (83.3%) died in hospital and 14 patients (77.8%) died in ICU, significantly higher than the non-cardiac arrest group (*P* < .001), which meant cardiac arrest was really a high-risk factor for hemodynamic decompensation PE. Interestingly, we found myocardial infarct took part 44.4% of cardiac arrest patients (*P* = .012) and it should be distinguished from PE due to the similar clinical signs. Urine output was lower (*P* = .017), which meant more end-organ hypoperfusion, and BUN (*P* = .003) and creatinine (*P* < .001) were both higher. Respiratory was higher (*P* = .003) and SpO2 (*P* < .001) was lower in cardiac arrest group.

**Table 4:** Baseline data and outcome of hemodynamic decompensation pulmonary embolism patients stratified by cardiac arrest complication

Variables	Non-cardiac arrest (N = 529)	Cardiac arrest (N = 18)	*P* value	Missing value
General information				
Age (years)	67.90 [56.45, 78.17]	59.03 [50.81, 65.64]	**.025**	0
Length of hospital stay (days)	14.51 [7.40, 25.39]	3.58 [0.54, 10.15]	**<.001**	0
Length of ICU stay (days)	5.39 [2.51, 12.23]	2.07 [0.42, 8.84]	**.01**	0
In-hospital death	172 (32.5%)	15 (83.3%)	**<.001**	
In-ICU death	126 (23.8%)	14 (77.8%)	**<.001**	
Laboratory test				
Lactate (mmol/L)	2.60 [1.60, 5.10]	7.35 [3.83, 13.70]	**<.001**	107
Anion gap (mEq/L)	17.00 [14.00, 20.00]	26.00 [21.00, 30.00]	**<.001**	1
BUN (mg/dL)	25.00 [16.00, 38.00]	38.50 [30.25, 60.00]	**.003**	1
Creatinine (mg/dL)	1.10 [0.80, 1.80]	2.50 [1.52, 4.80]	**<.001**	0
INR	1.40 [1.20, 1.80]	1.70 [1.40, 2.72]	**.042**	19
PT (s)	15.60 [13.50, 19.20]	18.50 [15.03, 29.43]	**.043**	18
Vital signs				
Respiratory rate	13.00 [10.00, 16.00]	16.50 [13.25, 21.75]	**.003**	1
SpO_2_ (%)	92.00 [88.00, 94.00]	80.50 [53.75, 83.00]	**<.001**	1
Complication				
Myocardial infarct	95 (18.0%)	8 (44.4%)	**.012**	0
Treatment				
Urine output on first day (mL/d)	1275.00 [733.25, 2050.00]	583.00 [70.50, 1552.50]	**.017**	24
Oxygen therapy			**.002**	0
None	24 (4.5%)	4 (22.2%)		
Non-invasive	132 (25.0%)	2 (11.1%)		
Invasive	373 (70.5%)	12 (66.7%)		
Severity score				
SAPSII	44.94 (15.47%)	56.06 (18.33%)	**.003**	0
SOFA	8.19 (4.23%)	11.94 (4.18%)	**<.001**	0
sPESI	2.60 (1.08%)	3.24 (0.83%)	**.016**	3

Continuous variables were presented as median and interquartile range, compared with Kruskal Wallis test; Categorical variables were presented as percentages, compared with Chi-square test. Two-tailed *P* value smaller than 0.05 was defined as statistical significance. Only significant variables were shown. Bold values mean statistical significance for *P* values.

BUN = blood urea nitrogen, ICU = intensive care unit, INR = international standard ratio, PT = prothrombin time, SAPSII = simplified acute physiology score II, SOFA = sequential organ failure assessment, sPESI = simplified pulmonary embolism severity index, SpO_2_ = percutaneous oxygen saturation.

## 4. Discussion

In this retrospective study, we determined that at ICU admission older age (>67 years), decreased hemoglobin (<11.3 g/dL) levels, elevated anion gap (>17 mEq/L) levels, elevated INR (>1.4), elevated respiratory rate (>13 times/min), decreased temperature (<37.3 °C), decreased SpO2 (<92 %) levels and the onset of cardiac arrest were significantly risk factors for in-Hospital mortality in PE patients with hemodynamic decompensation admitted to ICU.

Our results found that older age (>67 years) was positively related with in-Hospital mortality (Odd ratios [OR]: 1.016, 95% confidence interval [CI]: 1.001–1.033, *P* = .042) in patients with hemodynamic decompensation from the first multivariable model, which was generally in consistent with findings of the previous study. A retrospective study with a cohort of 310 hemodynamically unstable patients with PE in China suggested that the older age (>80 years) had a strong relationship with in-hospital all-cause mortality (OR: 18.7, 95% confidence interval [CI]: 1.7–207.5, *P* = .0169).^[9]^ However, the study defined age (>80 years) as a categorical variable and put it into the multivariable analysis. The different ethnic origins of our 2 studies could explain a part of the differences on the effects of aging. Another study including hemodynamic stable PE patients showed that older patients could lead to experience higher rates of right ventricle dysfunction and dilatation, atrial fibrillation during acute PE, which identified age as independent predictors of in-hospital mortality as well.^[10]^ Additionally, older age that could induce hypercoagulability of the organism, is known as one of the risk factors for the occurrence of VTE.^[11]^ Aging is also related with an elevated prevalence of chronic complications and alterations in immune system causing an augmented susceptibility to septicemia which causes or contributes to nearly one-third of all death in hospital.^[12,13]^ For PE patients with or without hemodynamic decompensation admitted to ICU, it suggested to study the direct effects of aging upon more subdivided age groups for all-cause in-Hospital mortality or PE-related mortality.

From our results, when the hemoglobin levels are <11.3 g/dL, decreased hemoglobin was independently associated with increasing risk of in-Hospital mortality (In first model: *P* = .025). According to the current anemia classification approach, patients with hemoglobin levels < 11.3 g/dL could be all divided into anemia group regardless of gender.^[14]^ And several studies concerning PE patients with anemia support our findings. A large scale research with a real-world populations demonstrated that VTE patients (comprising 38.9% PE patients and 61.1% deep venous thromboembolism patients) with anemia faced a higher risk of all-cause mortality and major bleeding, and severe anemia was related with a larger risk of adverse outcomes.^[15]^ Besides, the study with the multicentre registry in China also screened out that the anemia was one of risk factors associated with in-hospital all-cause mortality in hemodynamically stable PE patients (OR: 2.0, 95% CI: 1.3–3.0, *P* = .0020) though the multivariable analysis within hemodynamically unstable group excluded anemia as the independent factor.^[9]^ Additionally, a prospective study developed an original risk score to predict early major bleeding in acute PE including anemia (defined as hemoglobin < 12 g/dL) as one of the 3 assessment items and the score proportion of anemia item was highest, which identified anemia as the most powerful prognostic factor of early major bleeding.^[16]^ Though the mechanism of the relationship between anemia and poor prognosis remained unclear, anemia might hint at a tendency to hemorrhage or subclinical hemorrhage which require to be vigilant by clinicians. Moreover, it shown that higher INR was positively related with in-Hospital mortality from the results. Previous studies found that elevated INR while not on anticoagulation therapy appear to be at high risk of death among acute PE patients.^[17,18]^ Aggressive anticoagulation (INR > 2) could cause hematoma formation and higher risk of subsequent infection.^[19]^ However, despite the risk of bleeding or other complications, the use of anticoagulants therapy with adequate dosage and duration in the group with acute PE remains paramount and that INR is also useful as an auxiliary reference index.

Anion gap is an inexpensive and effective tool with wide clinal use which helps the detection of kinds of acid-base disorders, hematologic malignancies and intoxications.^[20]^ Many studies found that higher anion gap was related with an increased risk of all-cause mortality in critically ill patients with certain cardiovascular diseases such as cardiogenic shock, congestive heart failure, aortic aneurysm.^[21–23]^ Our study found that anion gap (> 17 mmol/L) was positively correlated with in-Hospital mortality in PE patients with hemodynamic instability (AUC: 0.664, 95% CI: 0.617–0.712). According to RCS results, there was almost a linear positive correlation between the levels of anion gap and the risk of in-Hospital death. Higher anion gap usually indicates there exists metabolic acidosis in the organism, which might be caused by the accumulation of lactate due to decreased oxygen delivery or defective oxygen utilization in PE patients or could be caused by acute kidney injury and lead to acid-base disturbance.^[24,25]^ In our study, ascending respiratory rate (> 13 times/minute) had a rather weak association with in-Hospital mortality (From first model, OR: 1.053, *P* = .043), which might potentially be caused by hypoxia and metabolic acidosis due to severe PE, or may be related with other respiratory complications in these patients. Another vital sign temperature was also an independent predictor for in-Hospital mortality screened together by the first and second model in our study (AUC: 0.612, 95% CI: 0.561–0.662). Although a prospective population-based study indicated that there was no obvious benefit of targeted temperature management in patients with PE related sudden cardiac arrest (Univariate OR: 1.7, 95% CI: 0.6–5.1, *P* = .35), it could be beneficial to maintain the body temperature of PE patients with hemodynamic decompensation above 37.3 celsius degrees according to our results, and also conduct other early aggressive cares may be helpful for prevention of secondary brain injury especially in PE patients with hemodynamic instability.^[26,27]^

A prospective study with hemodynamically stable PE patients has proven that the discriminatory power of the 2014 ESC algorithm for mortality could be improved by adding oxygen saturation in air of < 88% which the cutoff value of oxygen saturation was determined by the authors.^[28]^ Besides, the retrospective study with a large cohort of 7438 PE patients in China showed that partial pressure of oxygen (PaO2) (< 60 mm Hg) was an independent predictor for in-hospital all-cause mortality in the hemodynamically stable PE patients.^[9]^ These findings were basically inconsistent with our findings though the PE subgroups among us varied. In our study, there was almost the linear negative correlation between SpO2 and in-Hospital mortality from the RCS result in our study, and decreased SpO2 (<92 %) levels were significantly correlated with higher risk of in-Hospital death. These patients with SpO2 levels < 92 % might be affected by hypoxemia which is one of the features of serious PE and caused by the mismatch between ventilation and perfusion. Thus, it suggested that several oxygen therapy strategies such as high-flow oxygen, mechanical ventilation should be conducted timely to correct the hypoxemia. Additionally, avoiding excess oxygen during oxygen therapy (i.e., not starting supplemental oxygen when the SpO2 is 92% or 93%) seems sensible as per current guidelines to prevent the occurrence of hyperoxaemia.^[29]^

The heterogeneity of conditions causing high-risk PE could lead to the large variation of the mortality, among which cardiac arrest may be the most noteworthy inducement. The remarkable fact was that the mortality of high-risk PE patients in need of cardiopulmonary resuscitation was 65% while the mortality of high-risk patients with cardiogenic shock was 25%.^[30]^ According to our findings, cardiac arrest was the only categorical factor screened by 2 multivariable models and it greatly related with in-Hospital mortality (In the first model, OR: 5.777, 95% CI: 1.358–33.454, *P* = .028). Additionally, the data also showed that the incidence of myocardial infarct in cardiac arrest group was much higher than non-cardiac arrest (44.4% vs 18.0%, *P* = .012), which may greatly contribute to the reduced chance of successful rescue. Thus, clinical staff are suggested to implement additional early interventions to correct any reversible causes during patients’ resuscitation.^[31]^ The studies containing over 18,000 out of hospital cardiac arrest patients in French indicated that previous VTE and an initial non-shockable rhythm were significantly related with PE as the cause of cardiac arrest, and a weaker relationship with younger age.^[32,33]^ In consistent with their findings, the cardiac arrest group in our results also had younger age than non-cardiac arrest group (Median age: 59.03 years vs 67.90 years, *P* = .025).

Lactate (*P* < .001), CK-MB (*P* = .011), Troponin-T (*P* = .005) levels were significantly higher in the death group rather than the survival group in the baseline data of PE patients with hemodynamic decompensation admitted to ICU. Among them, lactate (AUC: 0.673) had better predictive value for in-Hospital death than CK-MB (AUC: 0.591) and Troponin-T (AUC: 0.605). In physiological view, lactate could be a non-specificity indicator of imbalance between tissue oxygen supply and demand, which may be caused by severe PE with overt or imminent hemodynamic decompensation. For one thing, lactate was a common prognostic marker in critically ill patients and strongly associated with increased mortality among ICU patients.^[34]^ For another thing, several studies also identified that acute PE patients with elevated plasma lactate levels were at high risk of short-term mortality, and the risk stratification ability of the 2019 ESC risk assessment could be improved when added lactate into the algorithm.^[35–37]^ CK-MB is one of well-known biomarkers for detecting myocardial injury, and a few studies have proven that high admission CK-MB levels was strongly associated with worse clinical outcomes and higher mortality rates in PE patients.^[38,39]^ Troponin-T is also one of noticeable indicators of myocardial injury, and in acute PE patients elevated troponin-T significantly correlates with right ventricular dysfunction and belongs to an independent predictor for short-term mortality.^[40]^ Additionally, the elevation of troponin-T shows a concentration-dependent relationship with acute outcome in acute PE patients, which suggested that the threshold of it for detection may vary within different PE subgroups.^[41]^ Unfortunately, due to the large number of missing values of these 3 indicators, they were not included in the multivariable regression for further analysis. Follow up large-scale studies could further explore their important values in acute PE patients with hemodynamic decompensation.

There are certain limitations in our study which should be mentioned. Firstly, some bias could not be avoided since this is a retrospective design. Secondly, it a pity that several remarkable markers such as lactate, troponin-T, CK-MB had plenty of missing values and the role of them could not be further determined in the PE patients with hemodynamic instability. Thirdly, the data were extracted from MIMIC-IV database and lack of the long-term outcomes, which also had the value of further analysis. Fourthly, our study only included biomarkers as prognostic factors. Therefore, prognostic factors in other forms, such as CHA2DS2-VASc Score, which is directly correlated with higher rates of mortality after PE, even in absence of atrial fibrillation,^[42,43]^ is not evaluated in our study, which need further research.

## 5. Conclusions

In conclusion, according to our analysis, at ICU admission older age, decreased hemoglobin levels, elevated anion gap levels, elevated INR, elevated respiratory rate, decreased temperature, decreased SpO2 levels and the onset of cardiac arrest were significantly risk factors for in-Hospital mortality in PE patients with hemodynamic decompensation admitted to ICU. Further prospective studies were suggested to explore the role of new prognostic criteria comprising these markers.

## Acknowledgments

The authors would like to thank the MIT for the management of the MIMIC-IV database.

## Author contributions

**Conceptualization:** Yunfeng Chu.

**Formal analysis:** Zhong Chen, Gaosheng Luo.

**Methodology:** Yanbin Peng, Zhong Chen.

**Visualization:** Gaosheng Luo, Yunfeng Chu.

**Writing – original draft:** Yanbin Peng, Zhongkai Luo, Bo Zhou.

**Writing – review & editing:** Siqi Zhu.
